# Rectal Foreign Bodies: A 10-Year Review of the National Electronic Injury Surveillance System

**DOI:** 10.7759/cureus.41471

**Published:** 2023-07-06

**Authors:** Nicholas W Sheets, Ian Waldrop, William C Carpenter, Emily Dubina, Bhani Kondal, Hayden Schultz, David Plurad

**Affiliations:** 1 Trauma and Acute Care Surgery, Riverside Community Hospital, Riverside, USA

**Keywords:** retained foreign object, consumer products, national electronic injury surveillance system, vibrator, sexual device, sexual health, foreign bodies, rectal, acute care surgery and trauma, lower gi or colorectal surgery

## Abstract

Introduction

Rectal foreign bodies may result in significant morbidity, potentially necessitating surgical intervention and ostomy creation. The sensitive nature of the diagnosis may lead to inaccurate patient history and possible delay in diagnosis. Currently, there is a paucity of large national studies addressing this diagnosis. Therefore, we present national data describing the demographics and incidence of patients presenting with rectal foreign bodies.

Methods

The National Electronic Injury Surveillance System (NEISS) was utilized to collect data regarding rectal foreign bodies. Ten years of data were collected from 2012 to 2021. Inclusion criteria focused on the diagnosis of “foreign body” coupled with pelvic and lower torso injuries. Exclusion criteria encompassed patients without a rectal foreign body clearly identified in the narrative. Patients were compared based on disposition as low severity (treated/examined and released or left without being seen) or high severity (treated and admitted/hospitalized, held for observation, or transferred to another facility). General descriptive and inferential analyses were performed regarding demographics and dispositions.

Results

A total of 1,806 emergency department (ED) visits were identified for inclusion. Patients ranged in age from 0 to 93 years, with a mean age of 30 years. The largest age group identified was 11-15 and 21-25 years. Most patients were male (64.6%) and white (47.1%). The most common foreign bodies were massage devices and vibrators (22.7%), jewelry (8.1%), pens and pencils (4.4%), fishing gears (activity, apparel, or equipment) (3.7%), and nonglass bottles or jars (2.6%). Patients requiring admission, observation, or transfer differed from those patients that were discharged from the ED by age, sex, race, and product involved.

Discussion

Rectal foreign bodies are a rare diagnosis with a growing incidence. Though the most common objects are massage devices and vibrators consistent with sexual stimulation devices, there are limited product guidelines for safe use. Further studies to help identify at-risk persons, safety precautions, and manufacturing guidelines may help prevent potential morbidity associated with rectal foreign bodies.

## Introduction

The incidence of retained rectal foreign bodies is increasing [1]. Previous studies have shown that older men are at higher risk [1-3]. Most retained rectal foreign bodies are sexual stimulation devices, which may pose a significant health risk [4]. While most patients can be treated with manual retrieval in the emergency department (ED), some require surgery including an exam under anesthesia or laparotomy [2,3]. Several small studies and few organizations have provided recommendations for treatment [3-5]. Currently, there is a paucity of large national studies describing this diagnosis. Therefore, we present national data to describe the demographics and incidence of patients presenting with rectal foreign bodies to identify potential areas for prevention.

## Materials and methods

The National Electronic Injury Surveillance System (NEISS) was utilized to collect data regarding rectal foreign bodies. The NEISS is a free-access public database created by the United States Consumer Product Safety Commission that collects nationwide data from approximately 100 participating hospitals regarding ED visits associated with consumer products [6]. Ten years of data were collected from 2012 to 2021. Inclusion criteria included cases identified by searching for the diagnosis of “foreign body” with pelvic and lower torso injuries. Additionally, a narrative search was performed to identify rectal foreign bodies. Exclusion criteria included patients without a rectal foreign body explicitly mentioned in the narrative as some patients had vaginal or other pelvic foreign bodies. General demographic information including treatment date, age, sex, race, diagnosis, body part, disposition, location of injury, and product were collected. Patients were further compared in regard to those who were treated/examined and released or left without being seen (low severity) against patients who were treated and admitted/hospitalized, held for observation, or transferred to another facility (high severity). General descriptive and comparative analyses were conducted by utilizing SPSS version 28.

## Results

**Figure 1:**
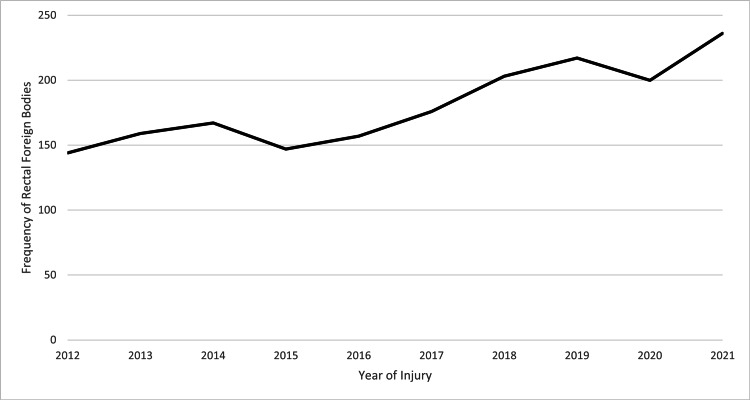
Frequency of Rectal Foreign Bodies by Year (2012-2021)

**Figure 2:**
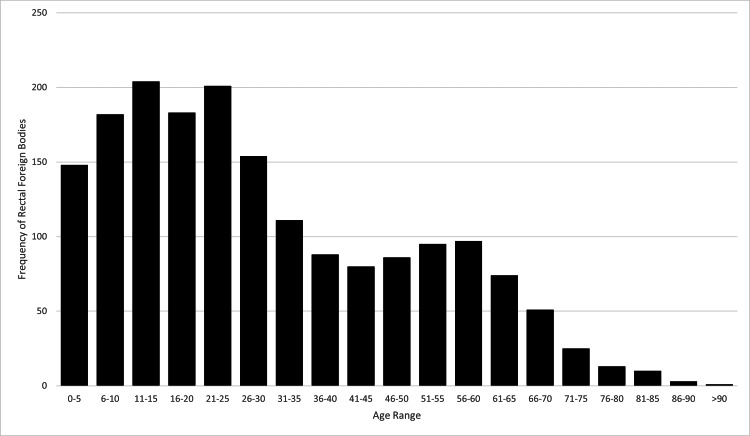
Frequency of Rectal Foreign Bodies by Age Range

**Figure 3:**
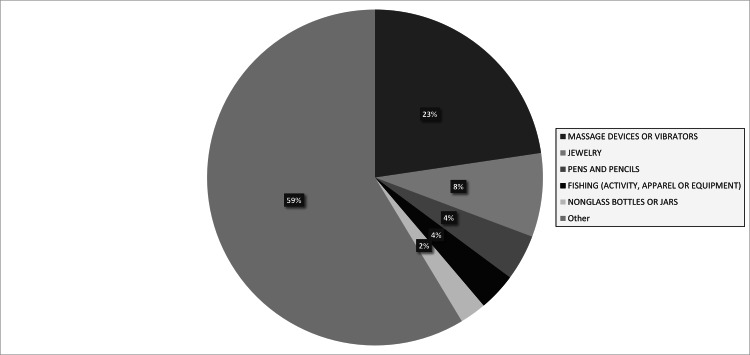
Rectal Foreign Body Product Incidence

There was a total of 135,844,348 ED visits between 2012 and 2021 included in the NEISS database. 1,806 ED visits were identified for meeting inclusion criteria (Table 1). The incidence of ED visits for rectal foreign bodies was 0.0013%. The incidence of rectal foreign bodies increased from 2012 (N=144) to 2021 (N=236) (Figure 1). Patients ranged in age from 0 to 93 years with a mean age of 30 years. The most represented age group was 11-15 and 21-25 years (Figure 2). Most patients were male (64.6%) and white (47.1%). Only 1.1% of patients recorded were positive for alcohol use; however, alcohol use was only recorded in 60.4% of patients as alcohol use was not reported by the NEISS database until 2019. The most common foreign bodies were massage devices and vibrators (22.7%), jewelry (8.1%), pens and pencils (4.4%), fishing gears (activity, apparel, or equipment) (3.6%), nonglass bottles or jars (2.6%), or other (58.5%) (Figure 3). Most patients were treated in the ED and then released (71.1%) with the remaining patients requiring admission (22.4%), observation (2.4%), left without being seen (2.0%), or transferred to another facility (2.1%). No ED deaths occurred.

**Table 1:** Demographics of Rectal Foreign Bodies Others*: rope or string, toys, bathtubs or shower fixtures, aerosol containers, balls, bags, batteries, marbles, paper products, golf equipment, dolls, plastic containers, glass bottles or jars, building sets, jewelry, sewing basket articles, crayons or chalk, candles, drinking glasses, screwdrivers, metal containers, cabinets and racks, flatware, kitchen gadgets, alcoholic beverages, plastic products, balloons, knives, manual cleaning equipment, flashlights, billiard products, non-electric razors, hair curlers, games and game parts, toothpicks, light bulbs, towels or cloths, soap, desk supplies, manicure/pedicure tools, coins, drapery, pipe, tie racks, tableware, telephone and accessories, glass tubing, pet supplies, nails/screws, electronics, drinking straw, computers, plastic wrapping, table tennis equipment, diapers, cigarette or pipe lighters, cleansing brushes, Christmas decorations, fishing equipment, portable food or beverage coolers, electric lighting equipment, bed or bedframes, toy vehicles, baseball equipment, vaporizers, ice picks, kitchen mixing bowls, scissors, thermometers, telescopes/binoculars/microscopes, and stereo accessories.

		Total	Low Severity	High Severity	p value
N		1806	1321	485	
Age Years (mean)		30	26.9	38.3	<0.05
Male n(%)		1166 (64.6)	770 (58.3)	396 (81.6)	<0.05
Race n(%)	White	851 (47.1)	606 (45.7)	247 (50.9)	<0.05
	Black/African American	260 (14.4)	219 (16.6)	41 (8.5)	<0.05
	Asian	17 (0.9)	12 (0.9)	5 (1.0)	0.81
	American Indian / Alaska Native	6 (0.3)	1 (0.1)	5 (1.0)	<0.05
	Unknown	582 (32.2)	421 (31.9)	161 (33.2)	0.59
Location n(%)	Home	827 (45.8)	562 (42.5)	265 (54.6)	<0.05
	Farm	2 (0.1)	1 (0.1)	1 (0.2)	0.46
	Street	7 (0.4)	6 (0.5)	1 (0.2)	0.45
	Public	72 (4.0)	58 (4.4)	14 (2.9)	0.15
	School	38 (2.1)	34 (2.6)	4 (0.8)	<0.05
	Sports	73 (4.0)	72 (5.5)	1 (0.2)	<0.05
	Unknown	787 (43.6)	588 (44.5)	199 (41.0)	0.19
Disposition n(%)	Treated/Examined and Released	1284 (71.1)	1284 (97.2)	-	-
	Treated and Transferred	38 (2.1)	-	38 (7.8)	-
	Treated and Admitted/Hospitalized	404 (22.4)	-	404 (83.3)	-
	Held for Observation	43 (2.4)	-	43 (8.9)	-
	Left Without Being Seen	37 (2.0)	37 (2.8)	-	-
Product n(%)	1st	Massage Devices/Vibrators 461 (22.7)	Massage Devices/Vibrators 273 (18.4)	Massage Devices/Vibrators 188 (34.4)	
	2nd	Jewelry 164 (8.1)	Jewelry 160 (10.8)	Nonglass Bottles or Jars 29 (5.3)	
	3rd	Pens and Pencils 90 (4.4)	Fishing Gears (Activity, Apparel or Equipment) 74 (5.0)	Pens and Pencils 29 (5.3)	
	4th	Fishing Gears (Activity, Apparel or Equipment) 74 (3.7)	Pens and Pencils 61 (4.1)	Bathtubs or Showers 18 (3.3)	
	5th	Nonglass Bottles or Jars 52 (2.6)	Gas, Air or Spring-Operated Guns 44 (3.0)	Bottles or Jars, Not Specified 16 (2.9)	
	Others*	1193 (58.5)	875 (58.8)	267 (48.8)	

Comparative analysis revealed significant variations in patient disposition based on age, sex, race, and product involvement (Table 1). Patients who required admission, observation, or transfer to another hospital (high severity) were more likely to be older (38.3 years vs 26.9 years, p<0.05), male (81.7% vs 58.3%, p<0.05), and white (50.9% vs 45.7%, p<0.05) when compared to patients who were released from the ED or left without being seen. Additionally, patients who were admitted, observed, or transferred had injuries related to massage devices or vibrators (34.4%), nonglass bottles or jars (5.3%), and pens and pencils (5.3%) while patients who were discharged or left without being seen had injuries related to massage devices and vibrators (18.4%), jewelry (10.8%), and fishing apparel or equipment (5.0%). There were significantly more massage devices or vibrator injuries in patients requiring admission, observation, or transfer.

## Discussion

The diagnosis of a rectal foreign body is rare but can result in significant morbidity. Studies evaluating the increasing incidence of rectal foreign bodies appear to be consistent across publications. Griffin et al. identified an increased incidence of rectal foreign bodies in their study from 1995 (2.41/1,000,000) to 2006 (5.46/1,000,000) [7]. Similarly, Dahlberg et al. noted an increasing incidence in their study using National Surgical Quality Improvement Program (NSQIP) data from 1.4 to 2.3 per 100,000 person-years between 2005 and 2016 in men and from 0.3 to 0.6 per 100,000 person-years in women [1]. The current study supports these findings, noting an increase from 144 patients in 2012 to 236 patients in 2021. This rising trend may be attributed to the growing industry of sexual stimulation devices and the increased use endorsed by the media [7].

Seasonality of rectal foreign bodies was suggested by Pathak et al. with most diagnoses occurring in October [8]. When evaluating the monthly incidence in the current study, most diagnoses occurred during July (N=108), September (N=103), August (N=102), and October (N=101). While a discreet seasonality is not identified in the presented study, this may offer an area for future research. 

The dominance of white male patients presenting with the diagnosis of rectal foreign bodies has been previously described and is consistent with the findings of this study [2]. We identified a bimodal distribution with peak ages at 11-15 and 21-25. The lowest incidence occurred at ages 41-45 and 70+ years. Griffin et al. reported injury rates were highest for ages 30-39 and 40-49 years, and lowest for those aged 60 and older [7]. As both studies were performed using the NEISS database, this may indicate a changing demographic age group. Our study additionally found that patients requiring admission, transfer, or observation were more often white (50.9% vs 45.7%, p<0.05) and were more likely to be older (38.3 vs 26.9, p<0.05) than patients who were released or left without being seen.

Most rectal foreign bodies are sexual devices [4]. Our study found 124 different retained products. The most commonly retained foreign bodies were massage devices or vibrators (40.8%) followed by jewelry (8.1%) and pens and pencils (4.4%). Our study additionally found that patients that required admission, observation, or transfer to another hospital were more likely to have a retained massage device or vibrator when compared to patients who were discharged from the ED or left against medical advice (34.4% vs 18.4%, p<0.05). Ploner et al. found that the motivation behind the use of rectal foreign bodies was most often sexual stimulation (35.8%), assault (5.2%), alcohol involvement (1.0%), and smuggling (0.6%) [4]. Shamir et al. found that most patients initially provided an inaccurate history but later admitted to using objects for sexual gratification once they had established a rapport with the clinician [5]. Retained products consistent with sexual stimulation devices offer an area for prevention such as guideline development. Currently, few guidelines are present when choosing sexual devices. Rullo et al. recommend choosing a device with a wide base or string when used anally [9]. Further device manufacturing guidelines or warnings can be addressed to help prevent most retained foreign body ER visits. Currently, sexual devices are not closely regulated. Federal involvement through an appropriate establishment such as the Food and Drug Administration (FDA) can assist in prevention through regulation.

Rectal foreign bodies can result in bodily harm. Griffin et al. found that most injuries were anorectal (78.1%); the management of which has been addressed by the American Association for the Surgery of Trauma (AAST) [7]. The AAST recommends non-operative management for partial-thickness injuries unless full-thickness injuries are identified [3]. This same study noted 33% of patients had full-thickness injuries [3]. The results of operative management are not benign. Brungardt et al. found that of the 109 patients in their study, 74.3% required an operation and 34.57% resulted in a colostomy [2]. Dahlberg et al. found that of their 85 patients, 74% of patients required admission due to unsuccessful removal in the ED, 9.4% required laparotomy, and 2.3% of patients required an ostomy [1]. 

This study has limitations due to the minimal amount of data collected from the ED that is available within the NEISS. Socioeconomic, treatment, inpatient, or outcome data other than ED disposition are not available. ED disposition may be a poor indicator of the severity of injury yet may allow some insight into hospital resource utilization. Furthermore, the retrospective nature of the data collection and the inherent variability in narrative descriptions can contribute to inaccuracies in the coding of the underrepresentation of cases. Future studies can examine specific product characteristics that lead to unfavorable outcomes so product development designs can aim to prevent retention.

## Conclusions

Rectal foreign bodies remain a unique diagnosis with a well-described demographic. Massage devices and vibrators, consistent with sexual stimulation devices, are the most commonly retained objects with few manufacturing guidelines. The treatment outcomes vary between studies; however, those patients requiring surgery are at risk for ostomy. The granularity of this study creates limitations such as types of injuries, treatments, and outcomes yet helps describe possible targets for prevention. Future studies are needed to further explore this topic and develop effective prevention strategies.
